# Supplementary material for: Global divergence in plant and mycorrhizal fungal diversity hotspots
Source: Nat Commun. 2025 Jul 31;16:6702. doi: 10.1038/s41467-025-60106-8 (PMC12314030; doi:10.1038/s41467-025-60106-8)
Supplement: Supplementary file 1 — Supplementary Information [file 41467_2025_60106_MOESM1_ESM.pdf]

Supplementary information for “Global divergence in plant and mycorrhizal fungal diversity hotspots”

Table S1: Results of the single-variable two-sided t-tests assessing whether ecoregion richness correlations within biomes (Figure S1) are significantly ($p < 0.05$) different from zero. Significant values are bolded. AM = arbuscular mycorrhizal and ECM = ectomycorrhizal.

	**AM**				**ECM**			
**Biome**	**t**	**df**	**p-value**	**Estimate (mean correlation)**	**t**	**df**	**p-value**	**Estimate (mean correlation)**
1: Tropical Moist Forests	−9.86	204	**<0.001**	−0.191	6.92	204	**<0.001**	0.128
2: Tropical Dry Forests	1.34	53	0.187	0.052	5.74	53	**<0.001**	0.220
3: Tropical Conifer Forests	−0.26	13	0.802	−0.020	2.04	13	0.062	0.144
4: Temperate Broadleaf Forests	−3.00	77	**0.004**	−0.091	4.23	77	**<0.001**	0.116
5: Temperate Conifer Forests	0.39	46	0.697	0.013	4.51	46	**<0.001**	0.159
6: Boreal Forests	2.44	25	**0.022**	0.120	5.32	25	**<0.001**	0.255
7: Tropical Grasslands	5.47	50	**<0.001**	0.236	5.58	51	**<0.001**	0.259
8: Temperate Grasslands	3.37	42	**0.002**	0.125	4.81	44	**<0.001**	0.193
9: Flooded Grasslands	1.41	21	0.172	0.121	5.69	20	**<0.001**	0.296
10: Montane Grasslands	2.36	44	**0.023**	0.131	5.94	44	**<0.001**	0.227
11: Tundra	2.70	28	**0.012**	0.112	2.97	28	**0.006**	0.142
12: Mediterranean Forests	−0.21	39	0.836	−0.008	5.91	39	**<0.001**	0.181
13: Deserts	7.88	93	**<0.001**	0.290	10.29	94	**<0.001**	0.314
14: Mangroves	−0.06	17	0.954	−0.007	5.52	18	**<0.001**	0.288

Table S2: Statistics of the overlap between plant and fungal richness hotspots (regions with richness values in the top 95th percentile). Hotspot regions were identified separately for each of the two richness geospatial layers available for each taxa (Van Nuland, et al. [1] and Mikryukov, et al. [2] for AM and ECM fungi, and Cai, et al. [3] and Sabatini, et al. [4] for plants). a) shows the hotspot “agreement” between the two geospatial layers available for each taxa – i.e., the percentage of total hotspot area (area identified in either of the layers; green area for plants and purple area for fungi in Figure 4) that was identified as a hotspot in both of the two layers (dark green and dark purple in Figure 4). b) shows pairwise comparisons of overlap between each of the individual layers (i.e., the comparisons show in Figure S7). The numbers represent the percentage of the fungal hotspot area that overlapped with plant hotspot area. Note that a) and b) are not directly comparable because a) shows the percentage of the total hotspot area (area of both layers) that is overlapping, whereas b) shows the percentage of only the fungal hotspot area that overlaps with plants.

	AM	ECM
**a) Hotspot agreement between same-taxa geospatial layers:**		
Fungi	7.30%	13.96%
Plants	18.12%	16.79%
**b) % of fungal hotspot area overlapping with plant hotspots**		
Van Nuland, et al. [1] and Cai, et al. [3]	13.73%	0.29%
Van Nuland, et al. [1] and Sabatini, et al. [4]	1.96%	0.12%
Mikryukov, et al. [2] and Cai, et al. [3]	1.40%	2.10%
Mikryukov, et al. [2] and Sabatini, et al. [4]	0.53%	0.17%

Table S3: Thresholds used to mask areas of high uncertainty from the analysis. All grid cells with values in the top 95th percentile were masked. See Methods for a more detailed description of uncertainty measures. AM = arbuscular mycorrhizal, ECM = ectomycorrhizal, IQR = interquartile range, CV = coefficient of variation (standard deviation divided by the mean), SD = standard deviation.

Taxa	Layer	Uncertainty measure	95th percentile threshold
Plants	Sabatini, et al. [4]	Percentage ratio between IQR and median (of 99 bootstrap replicates)	36.47
	Cai, et al. [3]	CV (of five modelling methods)	0.099
	Combined	CV of the two layers	0.152
AM fungi	Van Nuland, et al. [1]	CV (of 100 bootstrap replicates)	0.043
	Mikryukov, et al. [2]	SD (10-fold cross-validation)	0.383
	Combined	CV of the two layers	0.512
ECM fungi	Van Nuland, et al. [1]	CV (of 100 bootstrap replicates)	0.070
	Mikryukov, et al. [2]	SD (10-fold cross-validation)	0.443
	Combined	CV of the two layers	0.241

Table S4: Percentage of terrestrial land included in the analyses of plant–arbuscular mycorrhizal (AM) and plant–ectomycorrhizal (ECM) fungal hotspots and correlations after applying masks to remove areas with high uncertainty in the original alpha diversity predictions. See Methods for details of how uncertainty was defined. The approximate original total area excludes area classified as “Rock and Ice” in Antarctica and Greenland. Biome classification follows that of Olson, et al. [5].

Biome	Approximate original total area (million km^2)	% included in AM analyses	% included in ECM analyses
Global (all terrestrial area)	130.9	67.76	68.55
1: Tropical Moist Forests	19.49	90.66	90.11
2: Tropical Dry Forests	3.86	89.36	88.06
3: Tropical Conifer Forests	0.68	87.41	90.73
4: Temperate Broadleaf Forests	12.53	92.84	80.70
5: Temperate Conifer Forests	3.75	90.14	80.02
6: Boreal Forests	15.10	87.99	88.31
7: Tropical Grasslands	21.27	69.50	73.79
8: Temperate Grasslands	10.53	83.59	73.65
9: Flooded Grasslands	1.13	69.55	61.88
10: Montane Grasslands	4.75	43.67	41.05
11: Tundra	7.96	60.35	60.71
12: Mediterranean Forests	3.30	52.13	51.16
13: Deserts	26.22	21.09	32.67
14: Mangroves	0.35	57.16	54.02

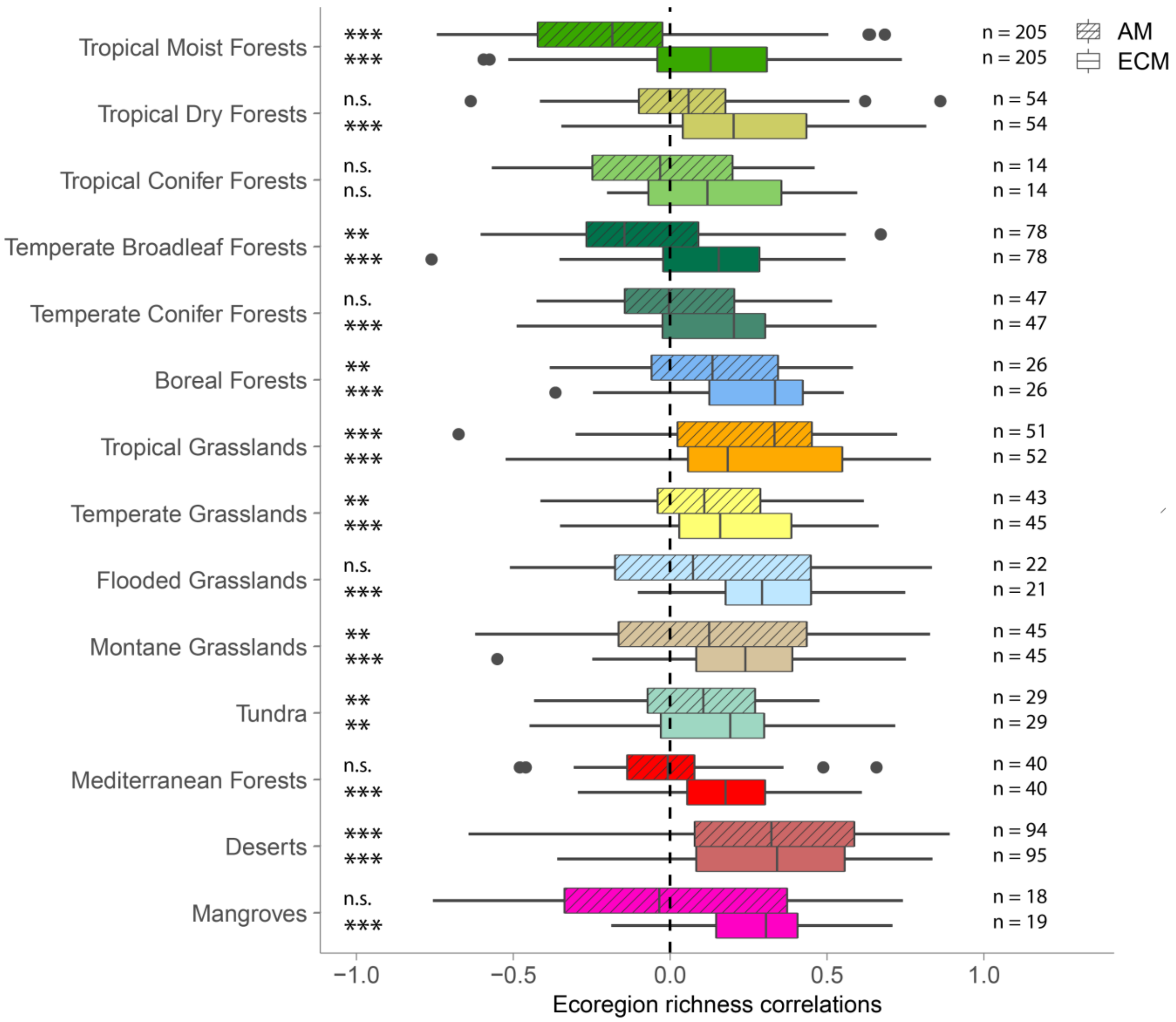

Figure S1: The distribution of arbuscular mycorrhizal (AM) and ectomycorrhizal (ECM) plant–fungal ecoregion correlations within each of the 14 biomes. n = the number of ecoregions included in each biome. Stars on the left-hand side show the results of single-variable t-tests assessing whether correlations are significantly different from zero (*** = $p < 0.001$, ** = $p < 0.01$, * = $p < 0.05$, n.s. = non-significant). See Table S1 for test statistics.

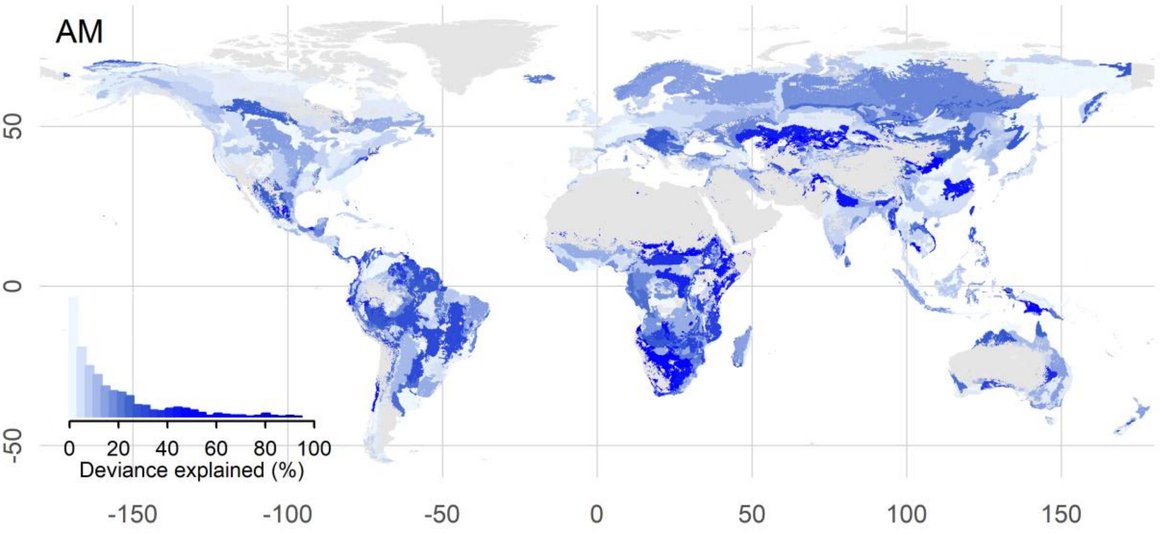

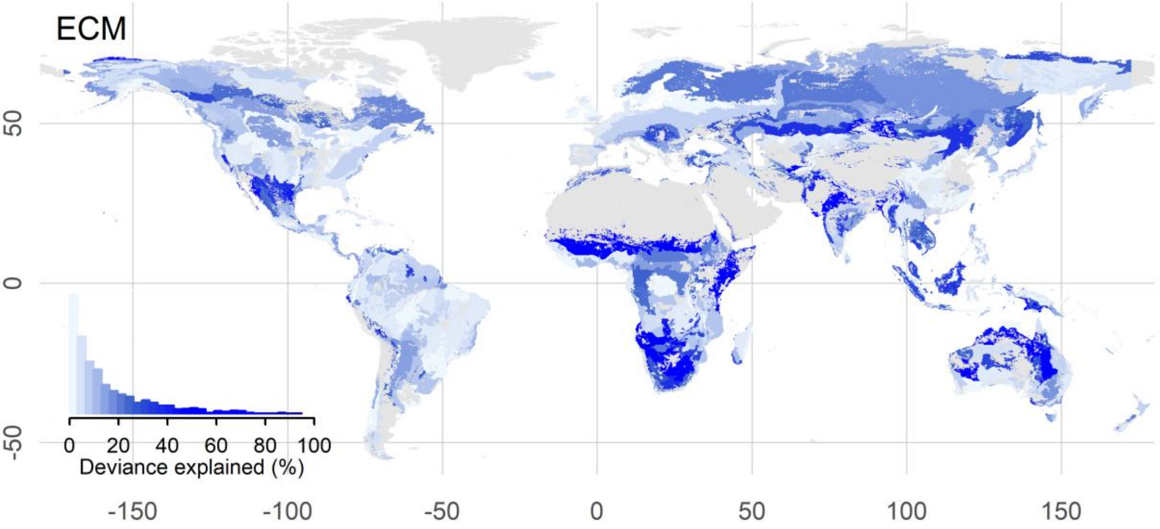

Figure S2: Relationships between plant and fungal richness from General Additive Models (GAMs) to account for non-linear relationships. Maps show ecoregion polygons[5] coloured by relationship strength (defined as deviance explained by the model). Grey areas were excluded from the analyses due to high uncertainty in the original alpha diversity predictions or where predictions for each taxonomic group by the different studies strongly disagreed (mostly based on the coefficient of variation between bootstrap model replicates; see Methods).

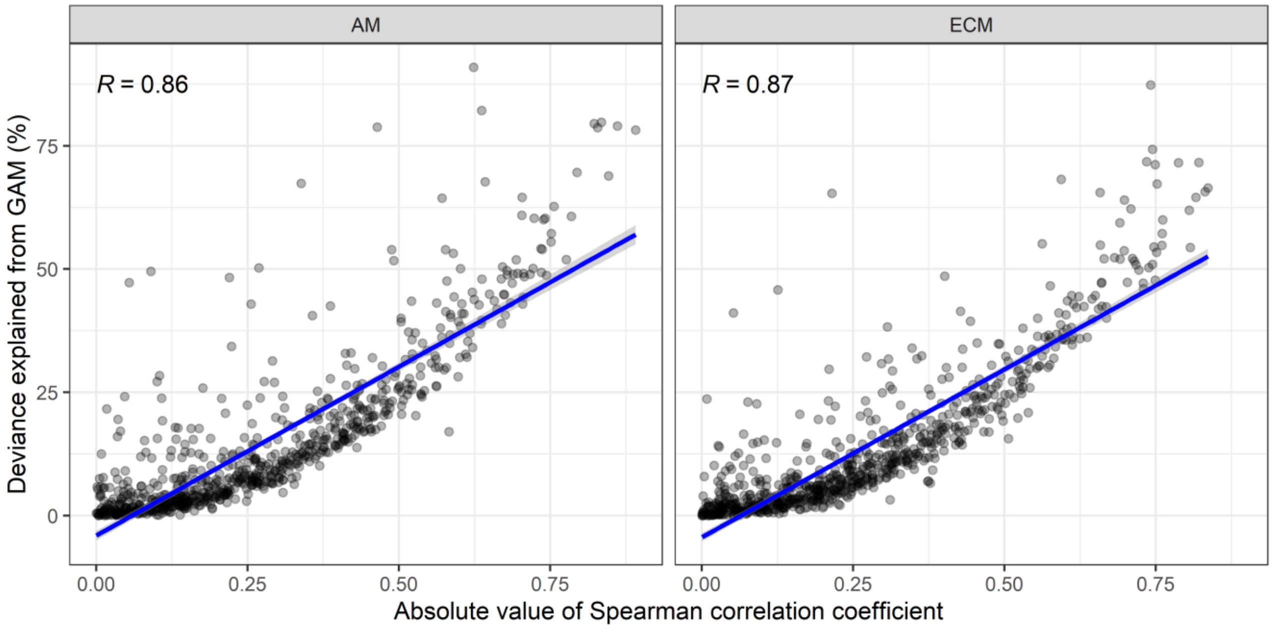

Figure S3: Relationship between the absolute value of plant and fungal richness Spearman correlation coefficients (from Figure 2) and deviance explained from GAMs modelling fungal richness as a function of plant richness (Figure S2) for each ecoregion. R shows the Spearman rank correlation between the two metrics. AM = arbuscular mycorrhizal and ECM = ectomycorrhizal.

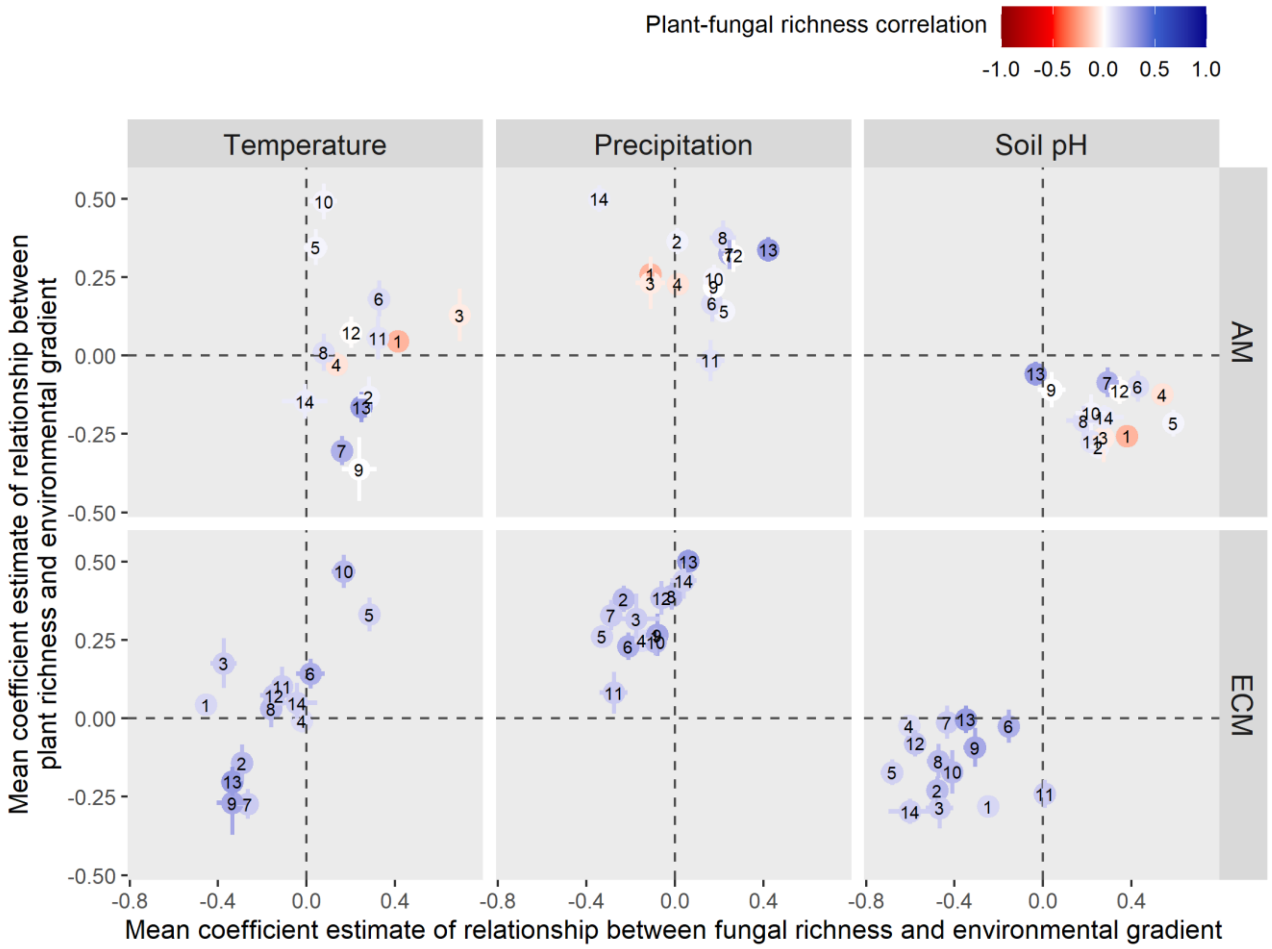

Figure S4: Mean and standard error of ecoregion coefficient estimates shown in Figure 3C for each of the 14 biomes, coloured by mean plant–fungal richness correlations. See Figure 3C and Methods for a description of how coefficient estimates were calculated. Values in the top right and bottom left segments are where plant and fungal richness respond similarly to environmental gradients (and therefore may be more likely to have positive (blue) plant–fungal richness correlations), and values in the top left and bottom right segments are where plant and fungal richness respond differently (and may be more likely to have negative (red) plant–fungal richness correlations). Point labels refer to 1: tropical moist forests, 2: tropical dry forests, 3: tropical conifer forests, 4: temperate broadleaf forests, 5: temperate conifer forests, 6: boreal forests, 7: tropical grasslands, 8: temperate grasslands, 9: flooded grasslands, 10: montane grasslands, 11: tundra, 12: mediterranean forests, 13: deserts, and 14: mangroves.

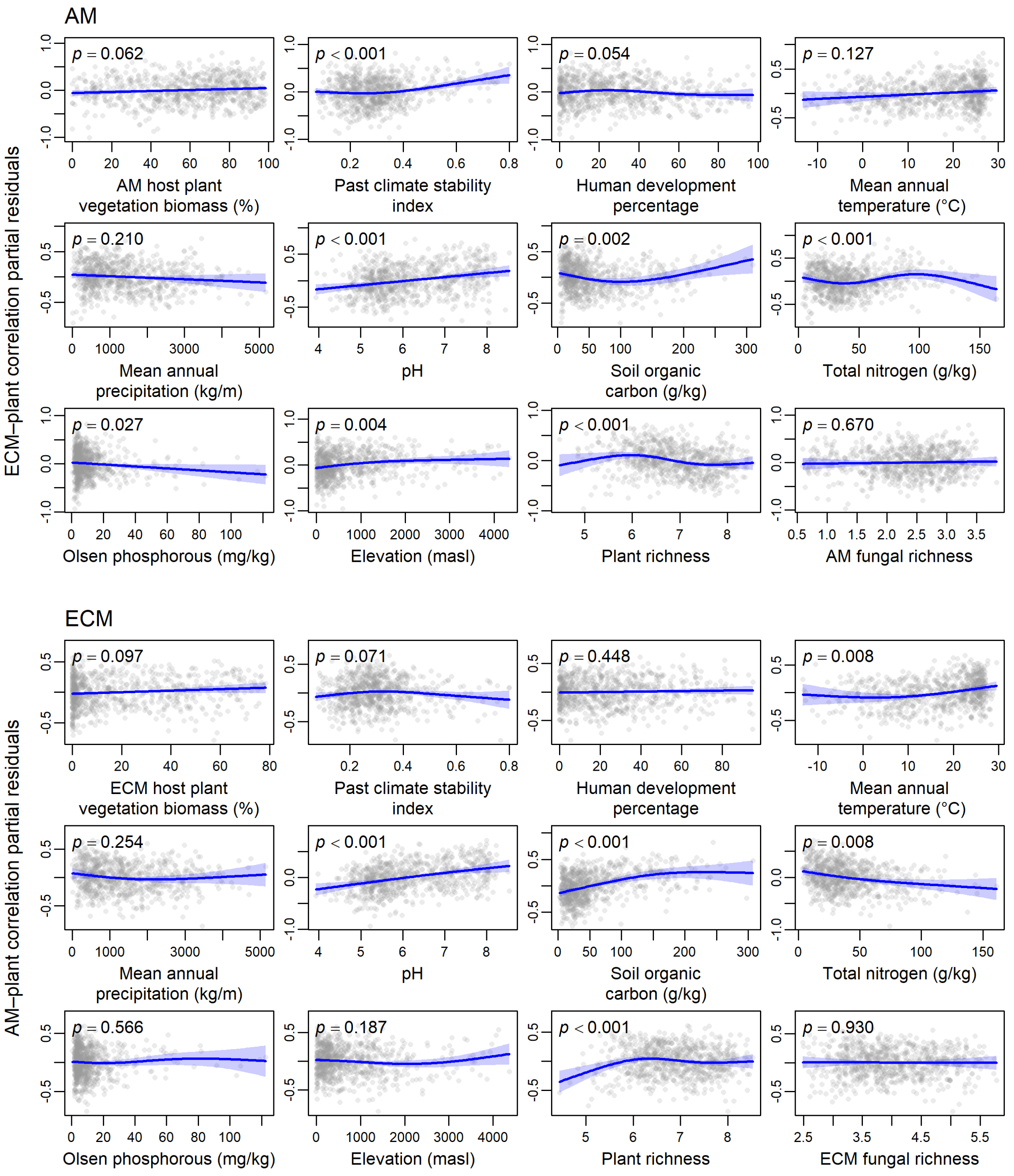

Figure S5: Partial residuals of all variables and covariates included in the GAMs testing the influence of host plant vegetation biomass, past climate stability, and human development percentage on fungal–plant richness correlations within ecoregions (those presented in Figure 3B, D and E). Shading shows one standard error of the fitted curves. For the AM (arbuscular mycorrhizal) model, deviance explained = 37.2%, adjusted $R^2 = 0.35$, and residual degrees of freedom = 727. For the ECM (ectomycorrhizal) model, deviance explained = 20.2%, adjusted $R^2 = 0.18$, and residual degrees of freedom = 732.

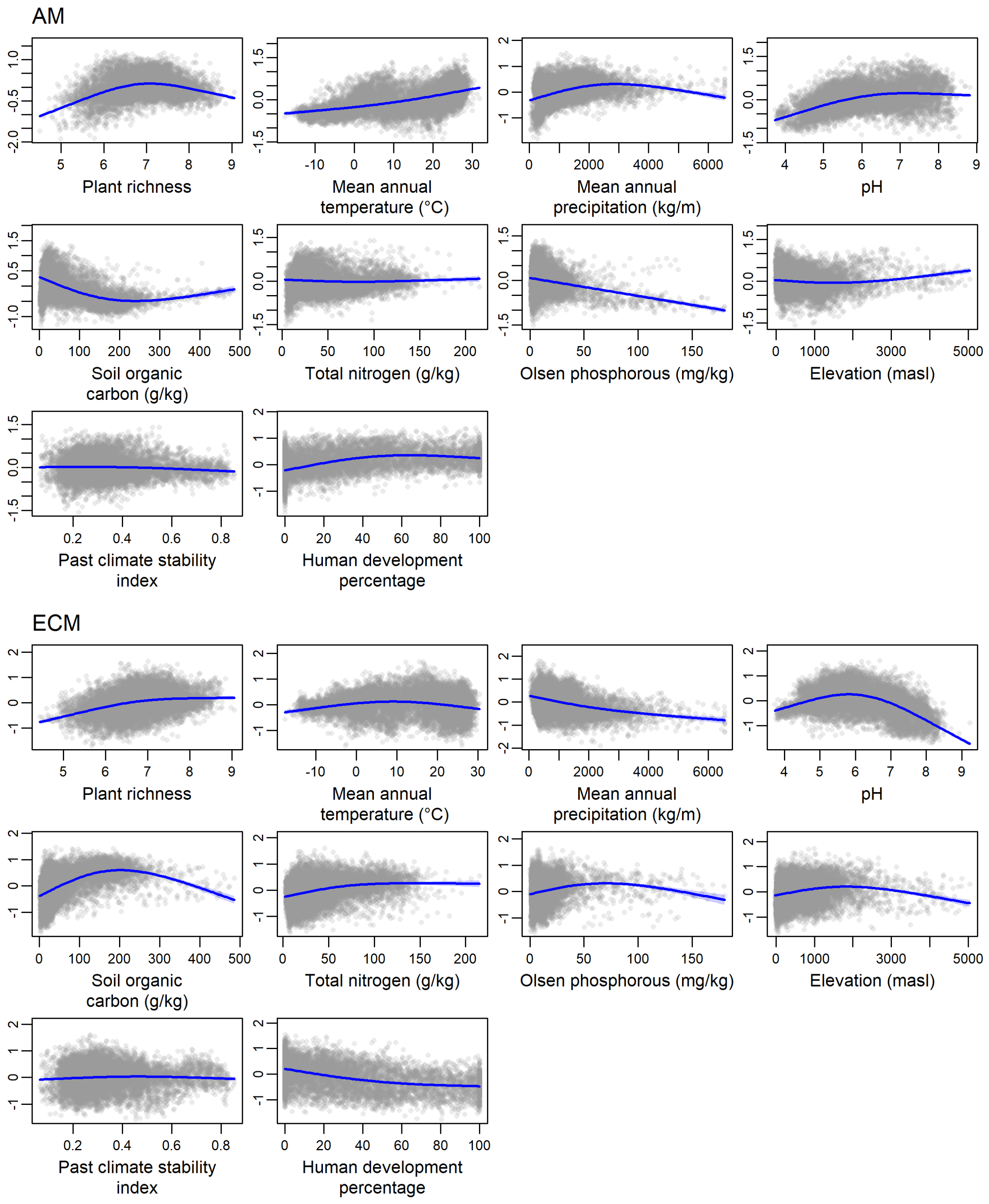

Figure S6: Partial residual plots of GAMs modelling arbuscular mycorrhizal (AM) fungal richness and ectomycorrhizal (ECM) fungal richness as a function of plant richness and other environmental covariates. The partial deviance explained by plant richness was then extracted and presented in Figure 3A. The percentage of vegetation biomass belonging to potential host biomass was not included as a covariate, because we were interested in the direct relationship between fungal and vascular plant richness irrespective of if plant species were potential hosts or not. Points are 10,000 randomly selected grid cells across the globe, shading shows one standard error of the fitted curves. For the AM (arbuscular mycorrhizal) model, deviance explained = 82.4%, adjusted R2 = 0.823, and residual degrees of freedom = 9,980. For the ECM (ectomycorrhizal) model, deviance explained = 73.3%, adjusted R2 = 0.733, and residual degrees of freedom = 9,979. p values are not shown because they were all highly significant due to the large number of points used.

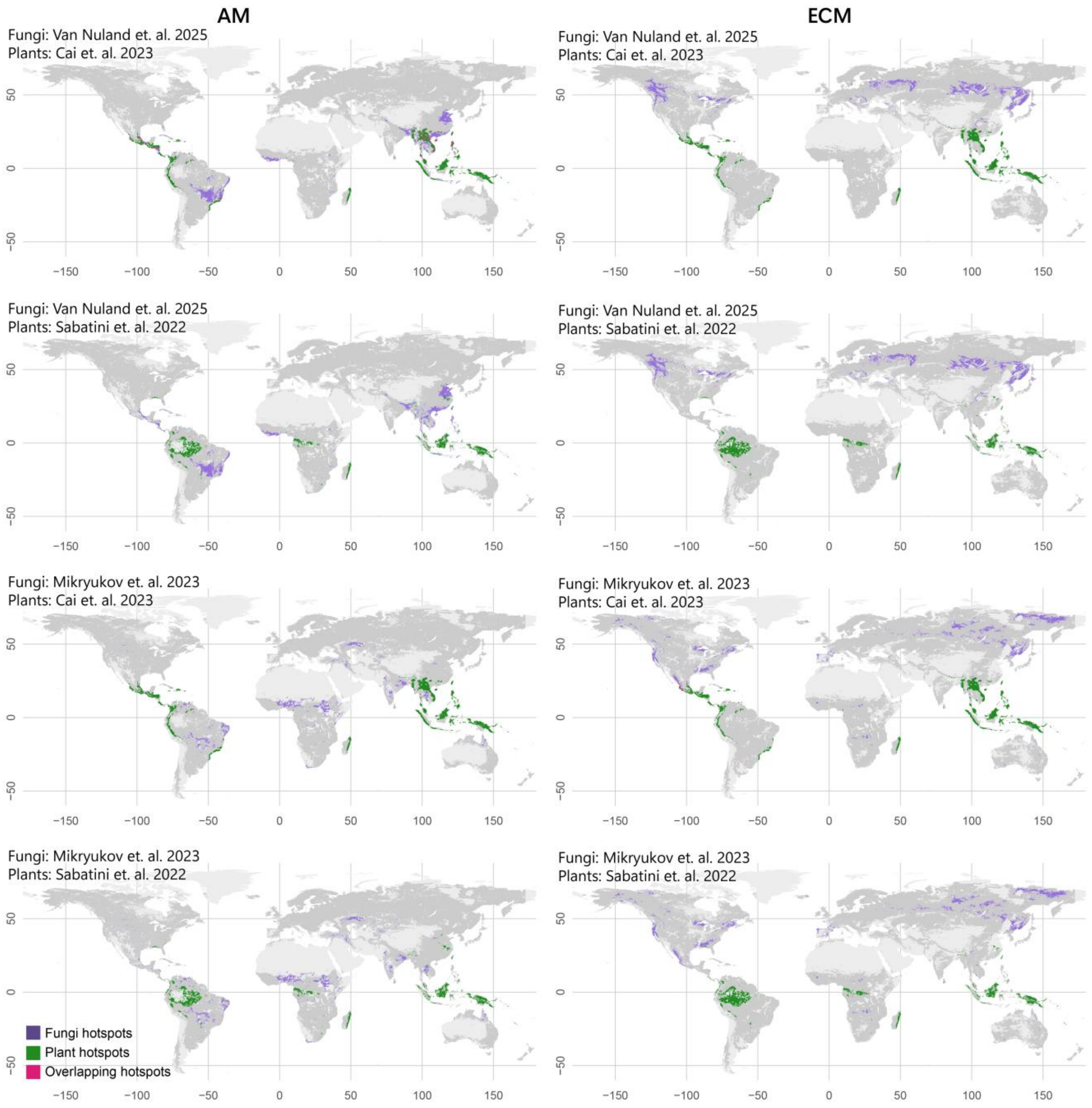

Figure S7: Hotspot (top 95th percentile) regions and plant–fungal hotspot overlap between each of the individual plant and fungal richness geospatial layers[1-4]. Percentage overlap statistics are provided in Table S2. Dark grey areas are those included in the analysis; light grey areas were excluded due to high uncertainty (see Methods). AM = arbuscular mycorrhizal and ECM = ectomycorrhizal.

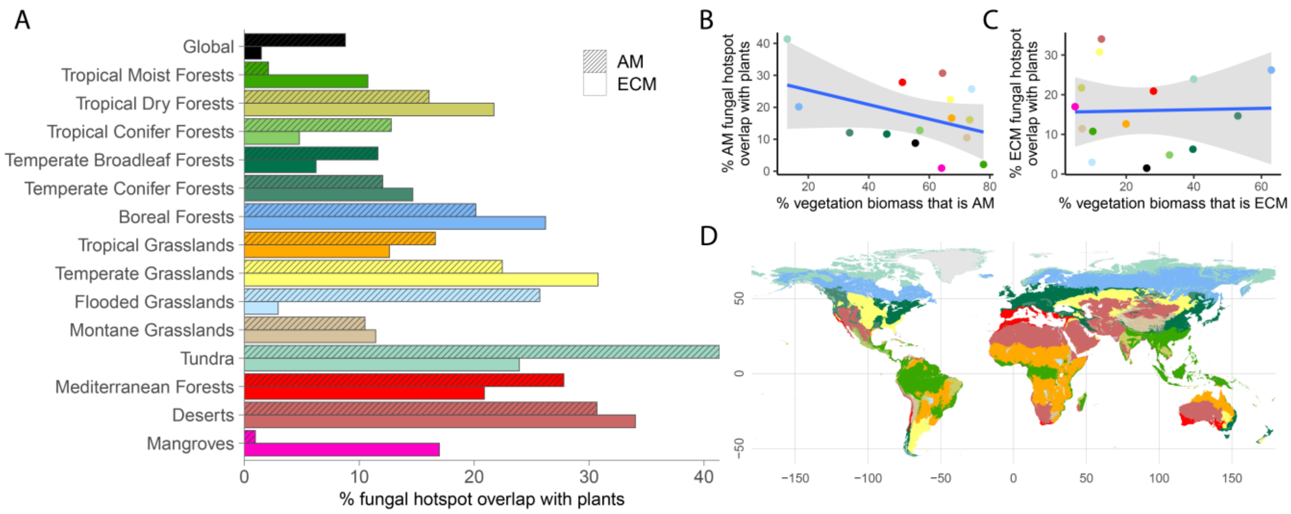

Figure S8: Plant–fungal hotspot overlap (top 95th percentile of richness predictions) re-calculated within each biome (A), and the relationship between hotspot overlap and the percentage of vegetation biomass belonging to the relevant plant host species (B and C). The relationship was non-significant for both AM and ECM models (AM: $t_{1,13} = -1.672$, $p = 0.118$, estimate = −0.227, 95% confidence interval = −0.52–0.07; ECM: $t_{1,13} = 0.105$, $p = 0.918$, estimate = 0.016, 95% confidence interval = −0.32–0.35). Colours in A to C correspond to biome regions shown in D[5,6]. Maps showing the distribution of hotspots within each biome are provided in Figures S9 and S10.

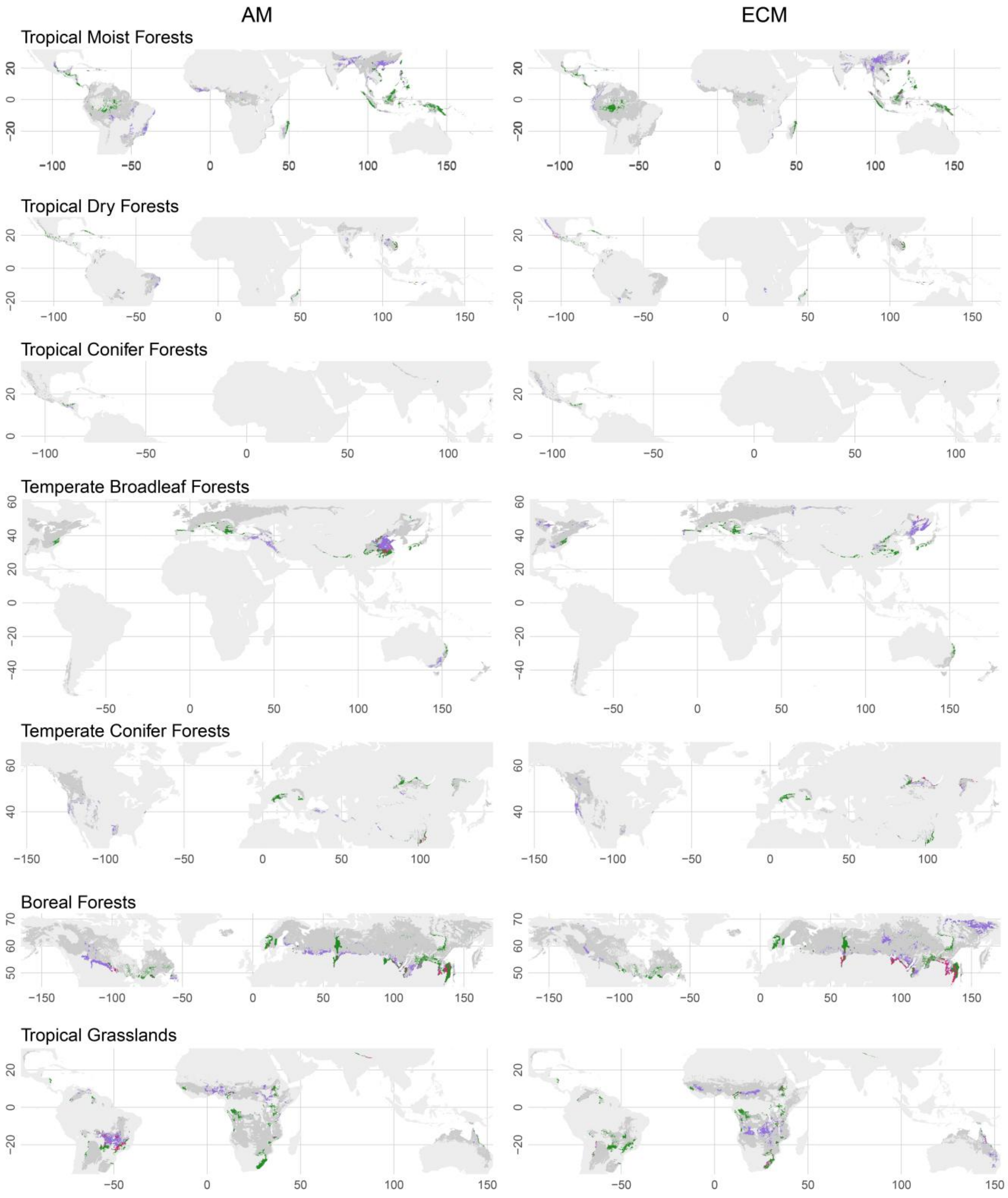

Figure S9: Plant (green) and mycorrhizal (purple) richness hotspots (top 95th percentile of richness predictions) within seven of the 14 biomes[5,6] (dark grey). Overlapping hotspot areas are shown in pink. Source data are the plant and fungal richness geospatial layers[1-4]. Remaining biomes are shown in Figure S10.

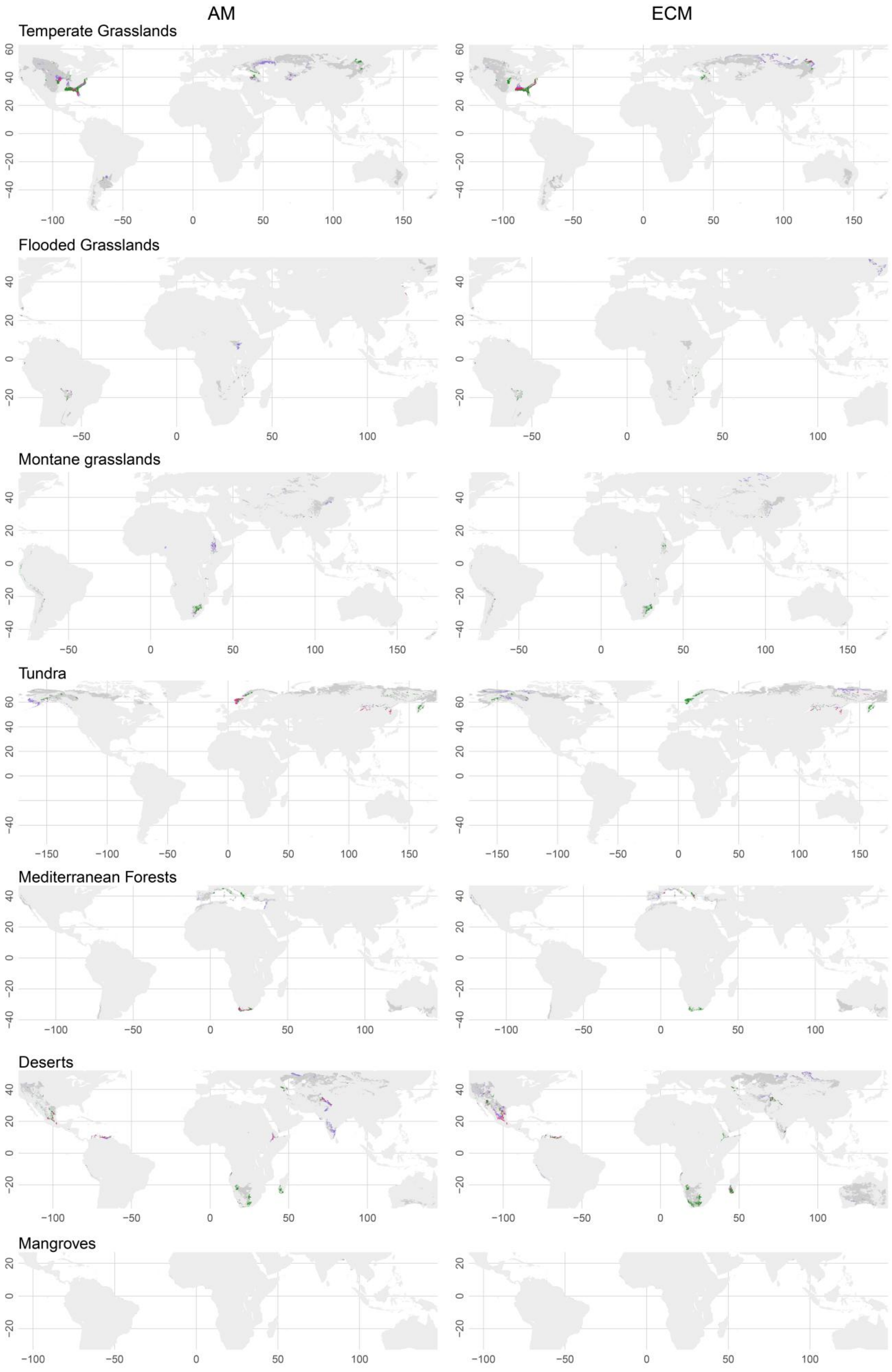

Figure S10: Plant (green) and mycorrhizal (purple) richness hotspots (top 95th percentile of richness predictions) within seven of the 14 biomes (dark grey). Overlapping hotspot areas are shown in pink. Source data are the plant and fungal richness geospatial layers[1-4]. Remaining biomes are shown in Figure S9.

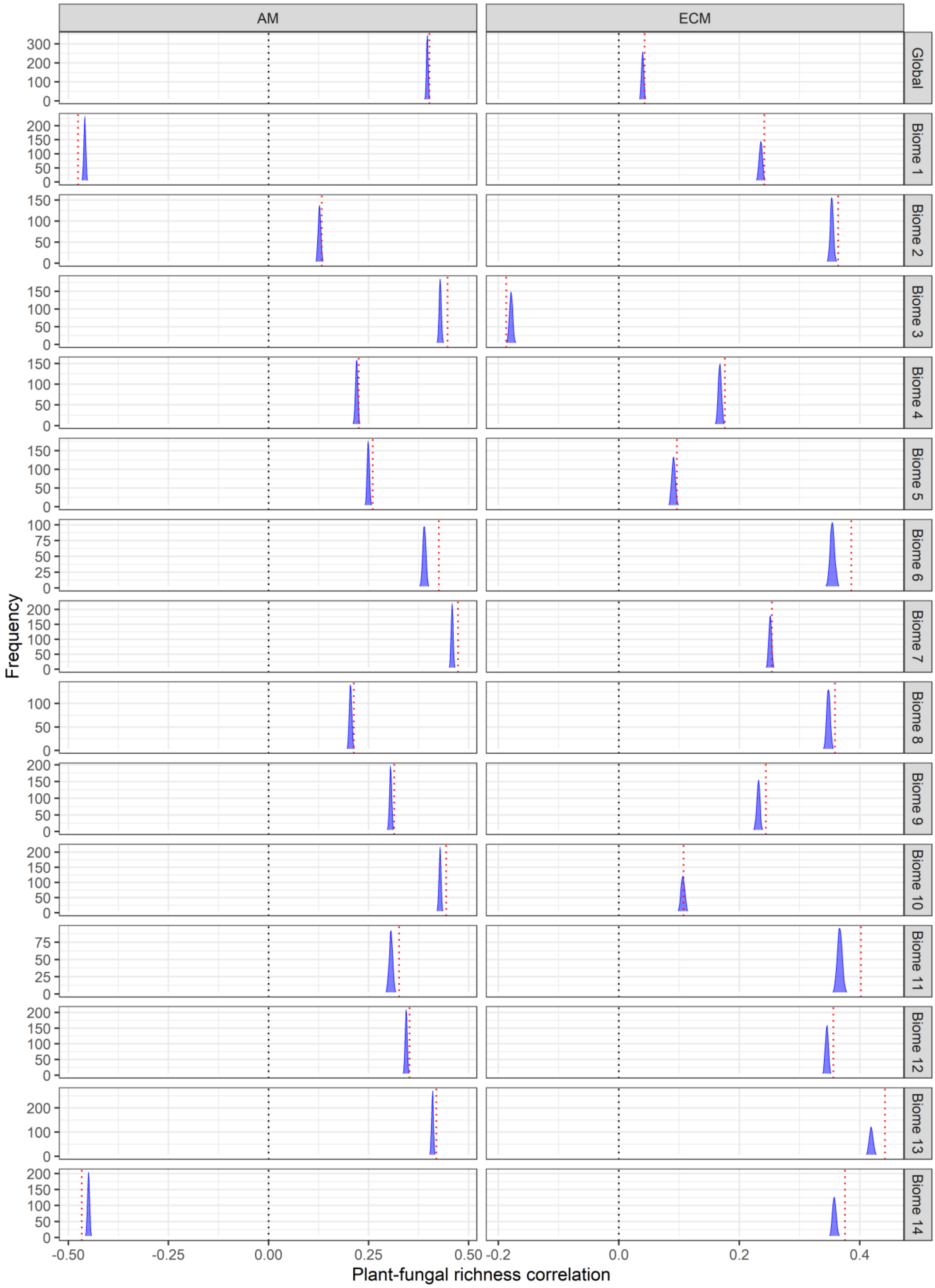

Figure S11: Analysis of how plant–fungal richness correlations change when accounting for prediction error in the geospatial richness layers. Red dashed lines are the original correlations between the combined plant and fungal geospatial layers used in the main analyses, and blue density plots show the distribution of 1,000 iterations of re-calculated correlations after adjusting for potential error (see Methods). Although correlations shift when error is accounted for, they do not change directions by shifting across the zero line (black dashed line).

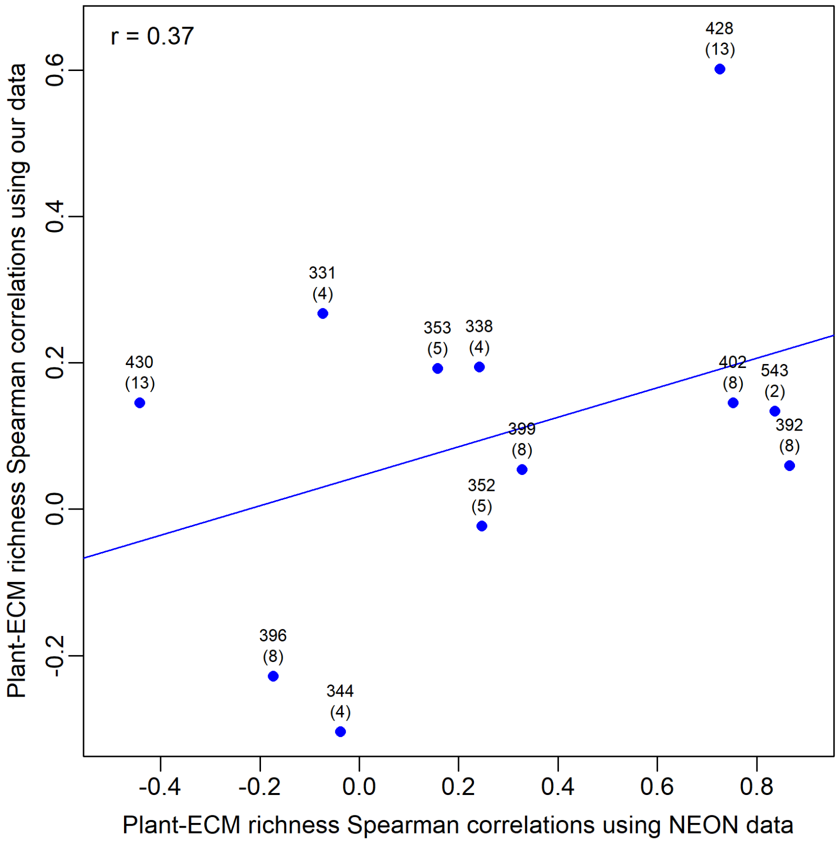

Figure S12: Comparison of plant–ECM fungal richness correlations (Spearman) calculated using ground-sourced data from the US NEON database (x-axis) and those calculated by our study using the modelled geospatial layers (y-axis). Only ecoregions where at least 10 plots were available in the NEON database were included. The blue line shows the linear relationship between the two methods ($t_{(1,10)}$ = 1.3, p = 0.23, estimate = 0.20, 95% confidence interval = −0.15–0.55), and the r-value is Pearson’s correlation coefficient. Labels show the ecoregion ID number, with the biome ID number in parentheses. Biome numbers refer to: 2 = tropical dry forests, 4 = temperate broadleaf forests, 5 = temperate conifer forests, 8 = temperate grasslands, and 13 = deserts. Ecoregion names can be found at https://ecoregions.appspot.com/.

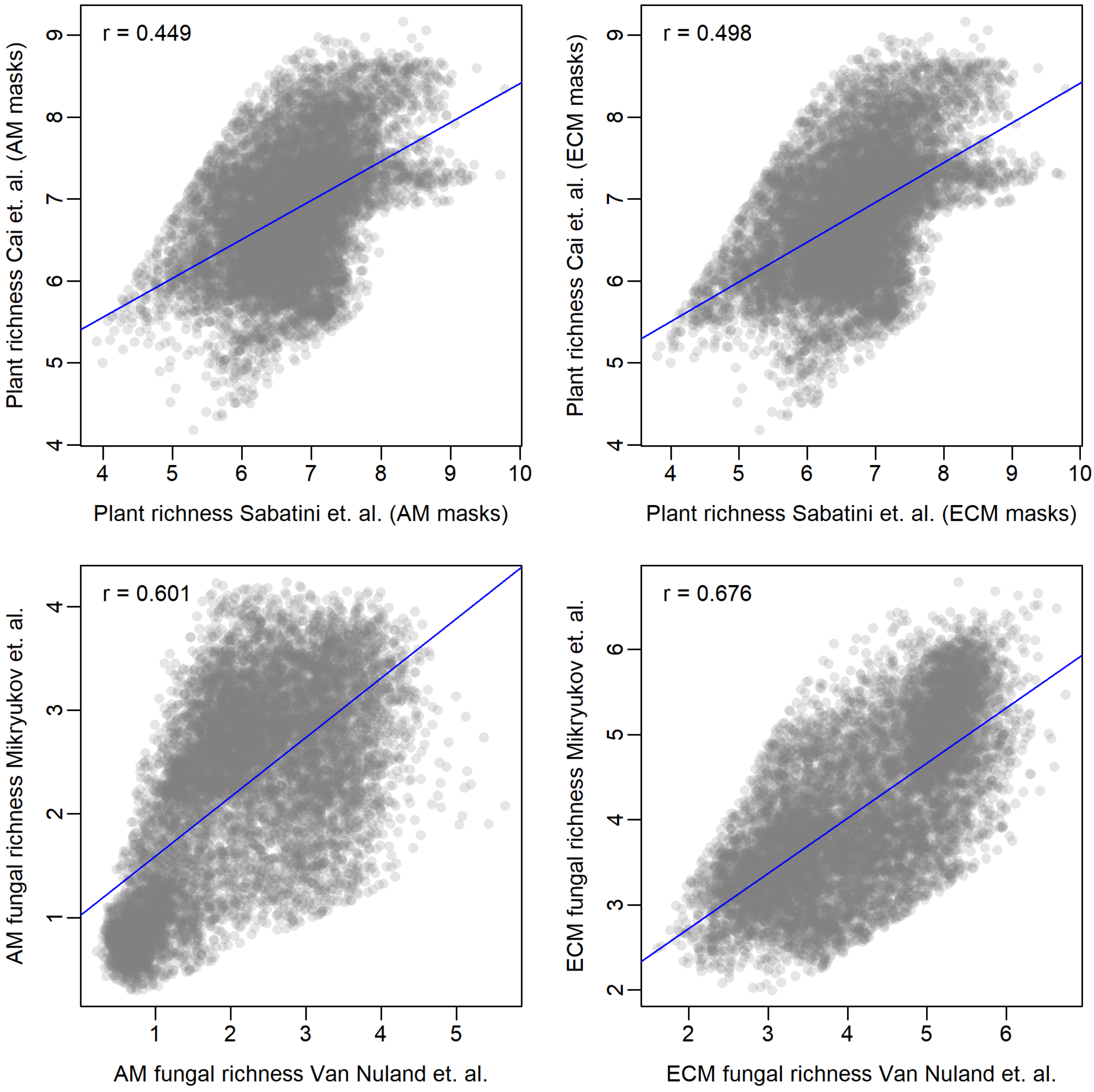

Figure S13: Relationships between richness values (log-transformed) predicted by the two geospatial layers available for each taxon, after applying uncertainty masks (see Methods). Points are values from 10,000 randomly selected grid cells across the globe. The relationship between the vascular plant layers is shown when the AM (arbuscular mycorrhizal) uncertainty masks were applied (for plant–AM analyses) and when the ECM (ectomycorrhizal) uncertainty masks were applied (for plant–ECM analyses). R-values are Spearman correlation coefficients, and blue lines show linear model trend lines (p values not shown because they were all highly significant due to the large number of points used).

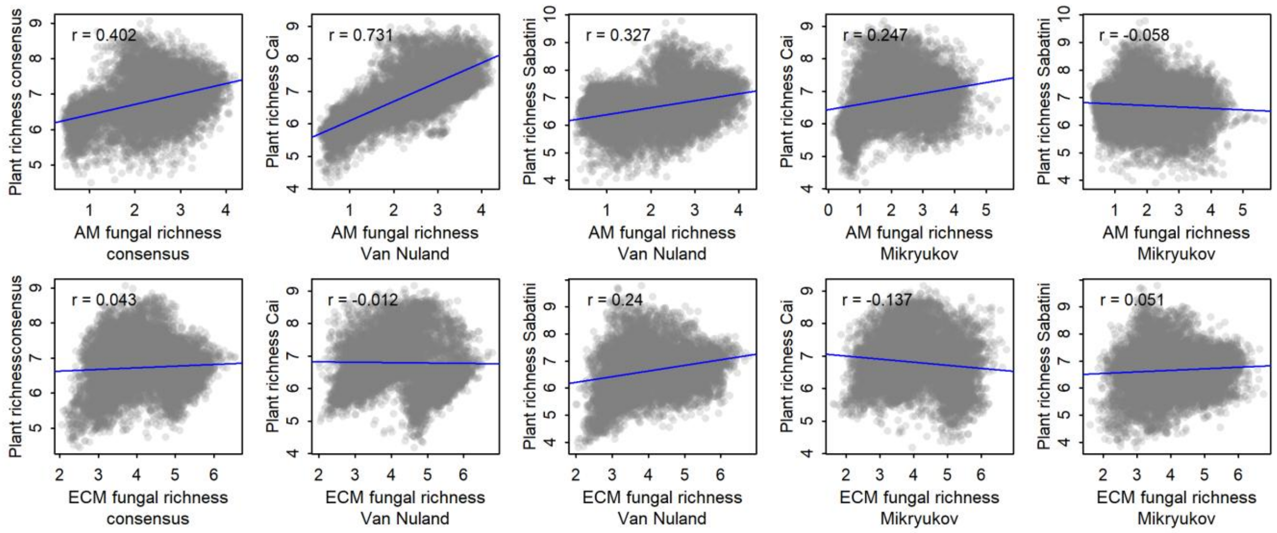

Figure S14: Pairwise global plant-fungal richness correlations of both the consensus richness layers used in the main analysis (see Methods) and the individual geospatial layers (plants: Cai, et al. [3] and Sabatini, et al. [4]; and fungi: Van Nuland, et al. [1] and Mikryukov, et al. [2]). Points are values from 10,000 randomly selected grid cells across the globe. R-values are Spearman correlation coefficients, and blue lines show linear model trend lines (p values not shown because they were all highly significant due to the large number of points used).

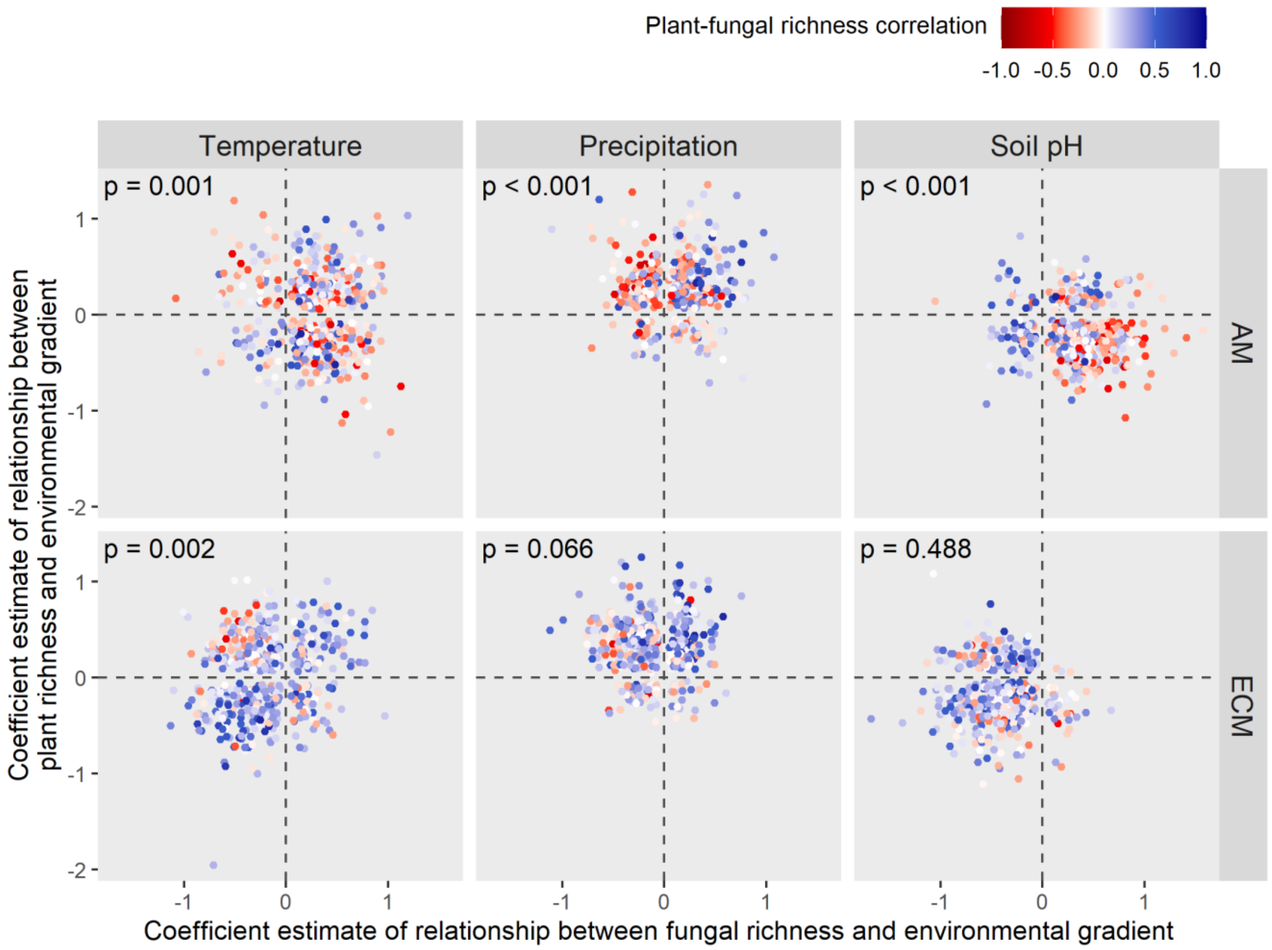

Figure S15: Analysis from Figure 3C but with ecoregions where predictor variables were correlated Pearson's r **> 0.85** removed (63 ecoregions for arbuscular mycorrhizal (AM) models and 64 for ectomycorrhizal (ECM) models). p-values show results of the chi-squared tests on the remaining ecoregions testing the hypothesis that positive plant–fungal correlations (blue) occur in ecoregions when plant and fungal richness respond similarly (top right and bottom left panel segments) to mean annual temperature, mean annual precipitation, and soil pH, and negative plant–fungal correlations (red) occur when they respond in opposite directions (top left and bottom right panel segments).

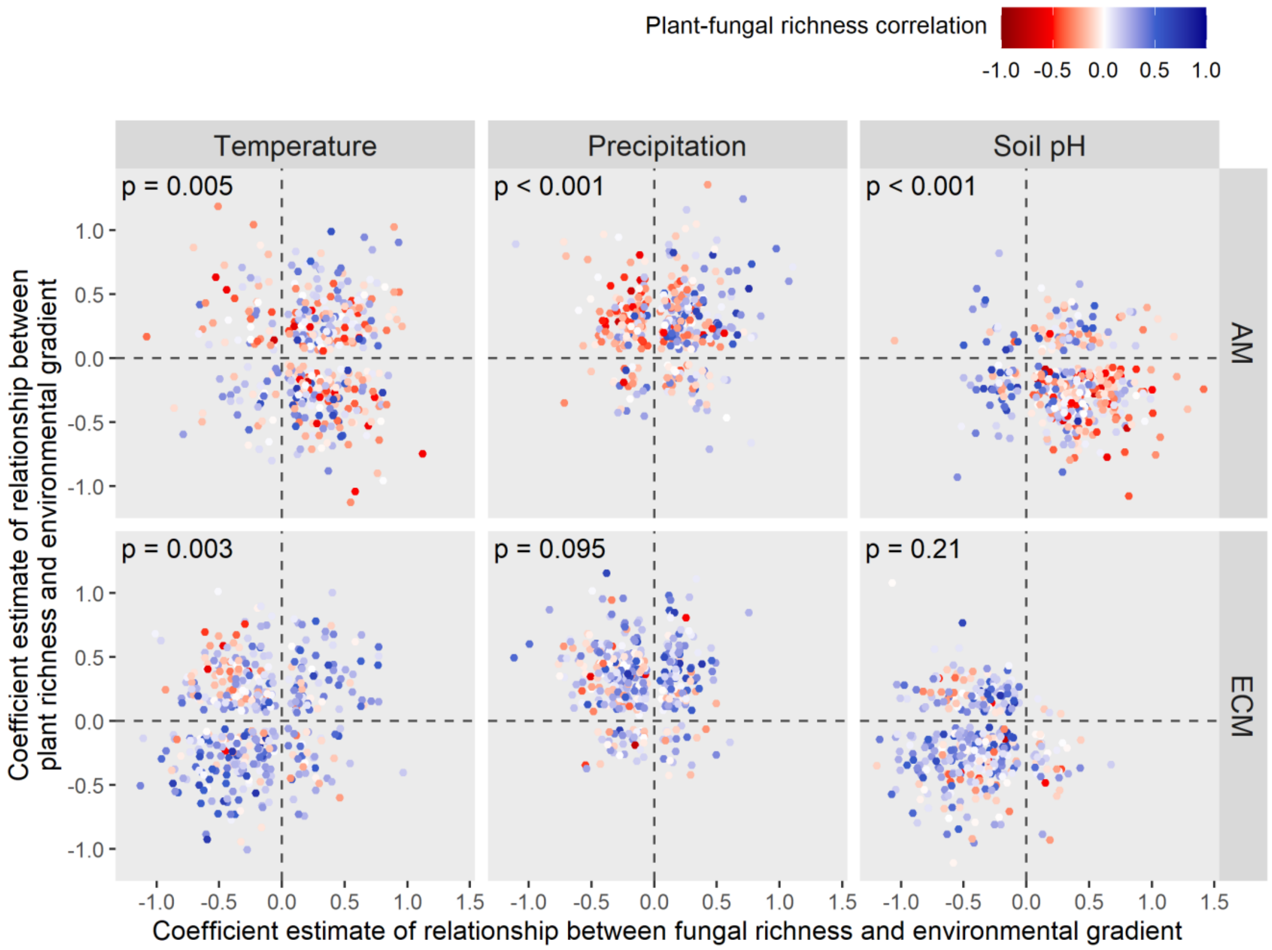

Figure S16: Analysis from Figure 3C but with ecoregions where predictor variables were correlated Pearson's r **> 0.8** removed (142 ecoregions for both arbuscular mycorrhizal (AM) models and ectomycorrhizal (ECM) models). p-values show results of the chi-squared tests on the remaining ecoregions testing the hypothesis that positive plant–fungal correlations (blue) occur in ecoregions when plant and fungal richness respond similarly (top right and bottom left panel segments) to mean annual temperature, mean annual precipitation, and soil pH, and negative plant–fungal correlations (red) occur when they respond in opposite directions (top left and bottom right panel segments).

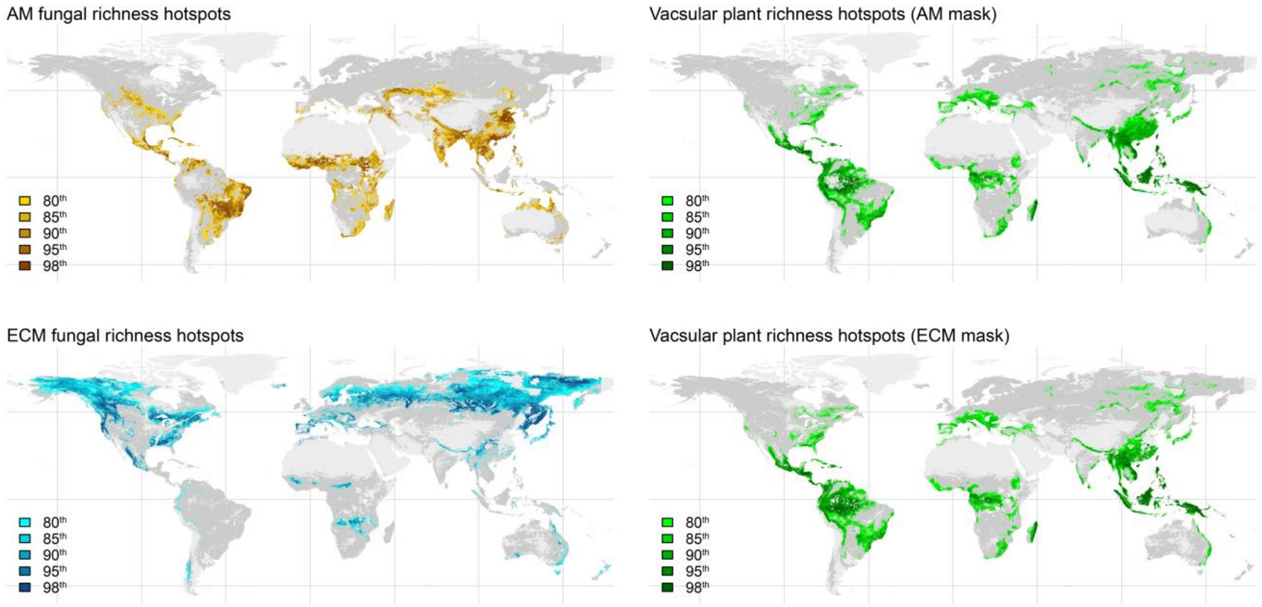

Figure S17: Plant and fungal richness hotspots identified using different percentile thresholds. Grid cells with richness values in the top 80th, 85th, 90th, 95th, and 98th percentiles are shaded progressively darker, to indicate how richness hotspots change when different thresholds are used. Hotspots are mapped individually for the four previously published alpha diversity geospatial layers[1-4] (and not the consensus richness maps), in a similar way to what is presented in Figure 4. Dark grey areas are those included in the analysis; light grey areas were masked due to high uncertainty in the original alpha diversity predictions or where predictions for each taxonomic group by different studies strongly disagreed. Different areas were excluded for AM (arbuscular mycorrhizal) and ECM (ectomycorrhizal) analyses, and so the plant richness hotspots are shown twice with the different uncertainty masks applied.

References

1 Van Nuland, M. E. *et al.* Global Hotspots of Mycorrhizal Fungal Richness are Poorly Protected. *Nature* **in press** (2025).

2 Mikryukov, V. *et al.* Connecting the multiple dimensions of global soil fungal diversity. *Science Advances* **9**, eadj8016 (2023). https://doi.org:doi:10.1126/sciadv.adj8016

3 Cai, L. *et al.* Global models and predictions of plant diversity based on advanced machine learning techniques. *New Phytologist* **237**, 1432-1445 (2023). https://doi.org:https://doi.org/10.1111/nph.18533

4 Sabatini, F. M. *et al.* Global patterns of vascular plant alpha diversity. *Nature Communications* **13**, 4683 (2022). https://doi.org:10.1038/s41467-022-32063-z

5 Olson, D. M. *et al.* Terrestrial Ecoregions of the World: A New Map of Life on Earth. *BioScience* **51**, 933-938 (2001). https://doi.org:https://doi.org/10.1641/0006-3568(2001)051[0933:TEOTWA]2.0.CO;2

6 Dinerstein, E. *et al.* An Ecoregion-Based Approach to Protecting Half the Terrestrial Realm. *BioScience* **67**, 534-545 (2017). https://doi.org:10.1093/biosci/bix014
