# Supplementary material for: Global divergence in plant and mycorrhizal fungal diversity hotspots
Source: Nat Commun. 2025 Jul 31;16:6702. doi: 10.1038/s41467-025-60106-8 (PMC12314030; doi:10.1038/s41467-025-60106-8)
Supplement: Supplementary file 3 — Reporting Summary [file 41467_2025_60106_MOESM3_ESM.pdf]

nature portfolio

Corresponding author(s): Laura van Galen

Last updated by author(s): Mar 24, 2025

Reporting Summary

Nature Portfolio wishes to improve the reproducibility of the work that we publish. This form provides structure for consistency and transparency in reporting. For further information on Nature Portfolio policies, see our Editorial Policies and the Editorial Policy Checklist.

Statistics

For all statistical analyses, confirm that the following items are present in the figure legend, table legend, main text, or Methods section.

n/a	Confirmed	
☐	☒	The exact sample size (*n*) for each experimental group/condition, given as a discrete number and unit of measurement
☐	☒	A statement on whether measurements were taken from distinct samples or whether the same sample was measured repeatedly
☐	☒	The statistical test(s) used AND whether they are one- or two-sided<br>*Only common tests should be described solely by name; describe more complex techniques in the Methods section.*
☐	☒	A description of all covariates tested
☐	☒	A description of any assumptions or corrections, such as tests of normality and adjustment for multiple comparisons
☐	☒	A full description of the statistical parameters including central tendency (e.g. means) or other basic estimates (e.g. regression coefficient) AND variation (e.g. standard deviation) or associated estimates of uncertainty (e.g. confidence intervals)
☐	☒	For null hypothesis testing, the test statistic (e.g. *F*, *t*, *r*) with confidence intervals, effect sizes, degrees of freedom and *P* value noted<br>*Give P values as exact values whenever suitable.*
☒	☐	For Bayesian analysis, information on the choice of priors and Markov chain Monte Carlo settings
☒	☐	For hierarchical and complex designs, identification of the appropriate level for tests and full reporting of outcomes
☐	☒	Estimates of effect sizes (e.g. Cohen's *d*, Pearson's *r*), indicating how they were calculated

Our web collection on statistics for biologists contains articles on many of the points above.

Software and code

Policy information about availability of computer code

Data collection	No data was collected
Data analysis	Data was analysed using R version 4.4.1 (open source). Code is available on Figshare at https://doi.org/10.6084/m9.figshare.28082948.

For manuscripts utilizing custom algorithms or software that are central to the research but not yet described in published literature, software must be made available to editors and reviewers. We strongly encourage code deposition in a community repository (e.g. GitHub). See the Nature Portfolio guidelines for submitting code & software for further information.

Data

Policy information about availability of data

All manuscripts must include a data availability statement. This statement should provide the following information, where applicable:

- Accession codes, unique identifiers, or web links for publicly available datasets
- A description of any restrictions on data availability
- For clinical datasets or third party data, please ensure that the statement adheres to our policy

The plant and fungal richness geospatial layers used in this study are available from Sabatini, et al. 28, Cai, et al. 27, Van Nuland, et al. 26, and Mikryukov, et al. 29. Data extracted from these layers used to produce the figures is available on Figshare at https://doi.org/10.6084/m9.figshare.28082948.

Research involving human participants, their data, or biological material

Policy information about studies with human participants or human data. See also policy information about sex, gender (identity/presentation), and sexual orientation and race, ethnicity and racism.

Reporting on sex and gender	NA
Reporting on race, ethnicity, or other socially relevant groupings	NA
Population characteristics	NA
Recruitment	NA
Ethics oversight	NA

Note that full information on the approval of the study protocol must also be provided in the manuscript.

Field-specific reporting

Please select the one below that is the best fit for your research. If you are not sure, read the appropriate sections before making your selection.

☐ Life sciences ☐ Behavioural & social sciences ☒ Ecological, evolutionary & environmental sciences

For a reference copy of the document with all sections, see nature.com/documents/nr-reporting-summary-flat.pdf

Ecological, evolutionary & environmental sciences study design

All studies must disclose on these points even when the disclosure is negative.

Study description	This study used previously published geospatial layers of species richness to analyse diversity correlation patterns at global, biome and ecoregion scales
Research sample	Data were from between 1,000 and 10,000 randomly selected grid cells of the geospatial layers (Sabatini et al. 2022, Cai et al. 2023, Van Nuland et al. 2025, Mikrykov et al. 2024)
Sampling strategy	Grid cells of geospatial layers were selected randomly
Data collection	No raw data were collected
Timing and spatial scale	No raw data were collected
Data exclusions	Some regions of the geospatial layers were excluded based on their prediction uncertainty. This was based on the uncertainty of the previously-published models used to predict richness (Sabatini et al. 2022, Cai et al. 2023, Van Nuland et al. 2025, Mikryukov et al. 2024). See methods for a full explanation.
Reproducibility	Data and code used to conduct the analysis and make the figures are made available so the study is fully reproducible
Randomization	Grid cells of geospatial layers were selected randomly
Blinding	Not applicable

Did the study involve field work? ☐ Yes ☒ No

Reporting for specific materials, systems and methods

We require information from authors about some types of materials, experimental systems and methods used in many studies. Here, indicate whether each material, system or method listed is relevant to your study. If you are not sure if a list item applies to your research, read the appropriate section before selecting a response.

Materials & experimental systems

n/a	Involved in the study	
☒	☐	Antibodies
☒	☐	Eukaryotic cell lines
☒	☐	Palaeontology and archaeology
☒	☐	Animals and other organisms
☒	☐	Clinical data
☒	☐	Dual use research of concern
☒	☐	Plants

Methods

n/a	Involved in the study	
☒	☐	ChIP-seq
☒	☐	Flow cytometry
☒	☐	MRI-based neuroimaging

Plants

Seed stocks	NA
Novel plant genotypes	NA
Authentication	NA
